# Light Trapping Induced High Short-Circuit Current Density in III-Nitride Nanorods/Si (111) Heterojunction Solar Cells

**DOI:** 10.1186/s11671-020-03392-z

**Published:** 2020-08-20

**Authors:** Ching-Wen Chang, Paritosh V. Wadekar, Hui-Chun Huang, Quark Yung-Sung Chen, Yuh-Renn Wu, Ray T. Chen, Li-Wei Tu

**Affiliations:** 1 Department of Physics and Center of Crystal Research, National Sun Yat-Sen University, Kaohsiung, 80424 Taiwan Republic of China; 2 Department of Electrical and Computer Engineering, The University of Texas at Austin, Austin, TX 78758 USA; 3 Research Center for Applied Sciences, Academia Sinica, Taipei, 11529 Taiwan Republic of China; 4 Department of Materials and Opto-electronic Science, National Sun Yat-Sen University, Kaohsiung, 80424 Taiwan Republic of China; 5 Department of Physics and Texas Center for Superconductivity, University of Houston, Houston, TX 77004 USA; 6 Institute of Photonics and Optoelectronics and Department of Electrical Engineering, National Taiwan University, Taipei, 10617 Taiwan Republic of China; 7 Department of Medical Laboratory Science and Biotechnology, Kaohsiung Medical University, Kaohsiung, 80708 Taiwan Republic of China

**Keywords:** III-nitride, Nanorod, Light trapping, Solar cell, Photovoltaic device, Plasma-assisted molecular beam epitaxy

## Abstract

An effective-area photovoltaic efficiency of 1.27% in power conversion, excluding the grid metal contact area and under 1 sun, AM 1.5G conditions, has been obtained for the p-GaN/i-InGaN/n-GaN diode arrays epitaxially grown on (111)-Si. The short-circuit current density is 14.96 mA/cm^2^ and the open-circuit voltage is 0.28 V. Enhanced light trapping acquired via multiple reflections within the strain and defect free III-nitride nanorod array structures and the short-wavelength responses boosted by the wide bandgap III-nitride constituents are believed to contribute to the observed enhancements in device performance.

## Introduction

The green energy development has become increasingly essential and light-emitting diode (LED) as well as solar cell industries have developed in a fast pace due to an ever-increasing energy crisis. Over the past few decades, III-nitride semiconductors have been successfully applied to LED devices [1–3], which have resulted in substantial commercial benefits. Presently, many scientists seek to exploit the research potential on III-nitrides for photovoltaic applications [4, 5]. Groups III-V nitride materials have many advantages for photovoltaic systems, such as a direct bandgap with a large absorption coefficient [4, 6], a wide bandgap range covering most of the solar spectrum via band engineering [4, 6, 7], high carrier mobility [7], and superior radiation resistance [8]. Based on these superb properties, several device structure designs are simulated, such as InGaN/Si tandem cells [9–14], hot carrier solar cells [15], Schottky-based solar cells [16–18], single [19–24] and multiple [25, 26] junction solar cells, as well as polarization effects on solar cell performance [9, 23, 27]. The simulations have predicted that InGaN/Si heterostructural tandem cells could have efficiencies as high as 21–36% [10, 11, 13] based on different simulation models. The power conversion efficiency (PCE) of InGaN homostructural tandem solar cell with four different In compositions is proposed to be 51% under 1-sun irradiance and 58% under 250-sun concentrated condition [26]. However, the issues of impurities and non-radiative recombination become increasingly significant under low-temperature InGaN thin film growth conditions [28–30]. The significant stacking faults and dislocation densities due to lattice mismatch lead to the decrease in carrier diffusion length and the limitation of solar cell PCE [31–34]. Therefore, numerous challenges remain for the realization of the potential capabilities of high-efficiency III-nitride photovoltaic devices.

In the past decade, many relevant research topics like high-In InGaN crystal growth methods on freestanding GaN substrate [34], p-type InGaN doping [35], quantum well designs [36–40], electrode designs [41–44], concentrator photovoltaics [37, 41, 45], intermediate band solar cells [46], and reflection-reduced structures [47–49] have been studied. Moreover, the nonpolar nitride-based solar cells were investigated on the polarization effect [50, 51]. Dahal et al. demonstrated a higher than 30%-In InGaN multiple-quantum-well solar cell operation at longer wavelengths (> 420 nm) [38] and illustrated a 3.03% efficiency under increased illumination intensity up to 30 suns [37]. Mori et al. investigated concentrator nitride-based solar cells [45] and addressed the highest PCE of 4% operated at a high light intensity up to 300 suns [41]. Even though several research groups provided different structural or optical designs and improved the growth techniques, the PCE of III-nitride solar cells did not advance much. On the other hand, Reichertz et al. demonstrated that tandem solar cells are feasible by epitaxially growing p-n junction GaN on p-n junction Si substrate [14]. Their results indicated that the Si substrate contributed long-wavelength efficiency while nitride contributed short-wavelength efficiency. Silicon substrates provide not only low-cost solution but also PCE enhancement and good thermal conductivity [52].

Usually, for solar cell growth, continuous film layers are grown on top of each other and this results in high dislocation density. However, when III-nitrides are grown in nanostructures, the bottom area in contact with the substrate is small hence threading dislocations are reduced and strain can also be minimal. Tessarek et al. reported that the dislocations of GaN nanorods vanished as the diameter goes down to 200 nm [53]. Therefore, as an alternative to film growth on silicon substrates, it would be a preferred choice to grow III-nitride nanorod solar cells to reduce the cost, to improve the crystal quality, and to enhance the cell efficiency. Also, nanorod/nanowire has a large capacity for photovoltaic applications because the photo-generated electrons can be collected more effectively before they recombined with holes due to a direct path to the electrodes and nanorod structures can improve light trapping for enhancing photon absorption [54, 55]. Several groups have demonstrated the photodetectors [56, 57], nanolasers [58, 59], nano-LEDs [60, 61], and photoelectrochemical water splitting applications [62] based on III-nitride nanorods [55]. Nonetheless, the demerit of nanorod solar cells is that photo-generated electron-hole pairs recombine at abundant carrier trapping centers due to surface defects. Moreover, the device fabrication processes of nanorod solar cells are more complicated than that of thin film devices. However, overcoming these issues mentioned above has resulted in an almost three-fold increase in PCE as shown by Wallentin et al. where the InP nanorod array has a 13.8% PCE from optimization of the nanorod diameter and the length of the top n-segment [54, 63]. Krogstrup et al. indicated that high short-circuit current density (J_sc_) was obtained in the single core-shell GaAs nanowire structures due to more than one order of magnitude light absorption enhanced by light concentration [64]. Wierer et al. [65], Cansizoglu et al. [66], and Nguyen et al. [31] demonstrated different types of nitride nanorod-array solar cells on GaN template and Si substrate. The comparison of recent nanorod/nanowire photovoltaic research is listed in Supplementary Information: Table S1. However, the photoelectric conversion contributions of different In content InGaN nanorod ensemble photovoltaic devices on low-cost Si (111) substrates have not been discussed systematically so far.

In this study, Mg:GaN/InGaN/Si:GaN III-nitride nanorod ensembles with 8% and 11% indium concentration were grown on n-doped Si (111) substrates by plasma-assisted molecular beam epitaxy (PA-MBE, Veeco EPI930). The structural properties and indium contents were estimated by high-resolution x-ray diffraction (HR-XRD, Bede D1) measurements. The fine structure of nanorods was analyzed by high-resolution transmission electron microscopy (HR-TEM, FEI E.O Tecnai F20 G2). The current density versus voltage (J-V) properties of nitride solar cells were discussed under 1 sun, AM 1.5G illumination (Newport 94023A). External quantum efficiency (EQE, Enli Technology Co., Ltd., QE-R3018) was measured to study the spectral response. The band diagram alignments and simulations were also investigated to explain the electron and hole transportation.

## Experimental Method

### Growth Technique

The growth of Si:GaN and Mg:GaN/InGaN/Si:GaN nanorods is based on the plasma-assisted molecular beam epitaxy (PA-MBE) technique. All samples were grown by a Veeco GEN930 PA-MBE system equipped with a 6N nitrogen plasma source (Veeco, UNI-Bulb). The n-type Si (111) substrate with a resistivity of 0.001–0.005 Ω cm was cleaned with acetone, isopropanol, and de-ionized water in an ultrasonic bath for 5 min at each step to remove residual organic contamination and then etched in a 48–51% HF:H_2_O = 1:5 solution for 5 min to remove the native oxide. After the chemical cleaning/etching process, the Si substrate was blown dry with nitrogen gas. The Si substrate was introduced into the buffer chamber and then transferred into the growth chamber by a magnetically coupled transfer arm. Prior to the nanorod growth, the substrate was thermally cleaned at 900 °C for 30 min to remove residual native oxide for obtaining a clean and ordered 7 × 7 reconstructed Si surface. The activated nitrogen atoms were generated by a plasma gun and its flux and purity were controlled via a high-resolution mass flow controller (HORIBA STEC, SEC-7320 M) and a nitrogen purifier (Entegris, CE35KFI4R). The high purity (6N or higher) Ga, In, Si, and Mg sources were provided by solid-source effusion cells. The group III metal and N_2_ plasma beam equivalent pressure (BEP) were measured with a beam flux gauge. By controlling the III/V flux ratios to N-rich condition, nanorods can be obtained. First, self-assembled Si:GaN nanorods were grown at 760 °C for 82 min. Desorption of InN is critical at raised temperatures because indium will evaporate from the sample surface. To retain indium in the nanorods, the metal-modulated epitaxy (MME) technique was utilized [67, 68]. MME involves periodic opening and closing of the metal shutters in order to modulate the metal fluxes, while the N_2_ shutter is kept open. For tuning In concentration, two different cycle times of In and Ga atoms impinged the substrate alternately for 20 s/30 s (sample B) and 30 s/30 s (sample C) with 50 periods at 550 °C. Finally, Mg:GaN layer was grown at 600 °C. The samples were grown under 9.25 × 10^−6^ torr active nitrogen BEP with plasma power 450 W, 2.42 × 10^−8^ torr In BEP, and 1.93 × 10^−8^ torr Ga BEP. In addition, the single-layer Si:GaN nanorods (sample A) was also prepared as a controlled group under the same condition.

### Device Fabrication

After the nanorod growth, the device fabrication process included the following steps. (1) The device area of 350 × 350 μm^2^ mesa was defined by etching down to the n-type Si with tetrafluoromethane (CF_4_) based on the reactive-ion etching technique (Advanced System Technology, Cirie-200) using photoresist (Microchemicals GmbH, AZ1400) as a mask. (2) An ultrasonic bath with de-ionized water was used to clean out loose nanorods from the device except those of the mesa area. (3) Immerse the sample in (NH_4_)_2_S at 60 °C for 1 min to passivate the nitride surface for native oxide suppression and non-radiative recombination reduction [69–73]. (4) A 100-nm indium tin oxide (ITO) thin film was deposited on top of the nanorods to serve as the Mg:GaN ohmic transparent contact by sputtering (Advanced System Technology, Psur-100HB) accompanied by photolithography (M&R Nano Technology, AG350-6B) and lift-off techniques. (5) Multilayer Ti/Al/Ti/Au (20 nm/300 nm/20 nm/50 nm) grid metal contacts on the ITO film and on the n-type Si substrates were fabricated by e-beam evaporation (Advanced System Technology, Peva-600E) using photolithography and lift-off techniques. (6) All grid metal contacts were annealed by rapid thermal annealing system (Advanced System Technology, FA04) for 30 s in nitrogen at 800 °C to obtain ohmic contacts.

### TEM Sample Preparation

To further study the crystal structure, individual nanorods of samples B and C were extracted by sonication in ethanol. After 30 min of sonication, a few drops of the ethanol solution were applied to a copper grid (Ted Pella) and the ethanol was evaporated at room temperature. Before the measurements, the sample was baked at 150 °C to remove free organic solvents.

## Results and Discussion

### Morphological and Structural Properties

**Fig. 1:**
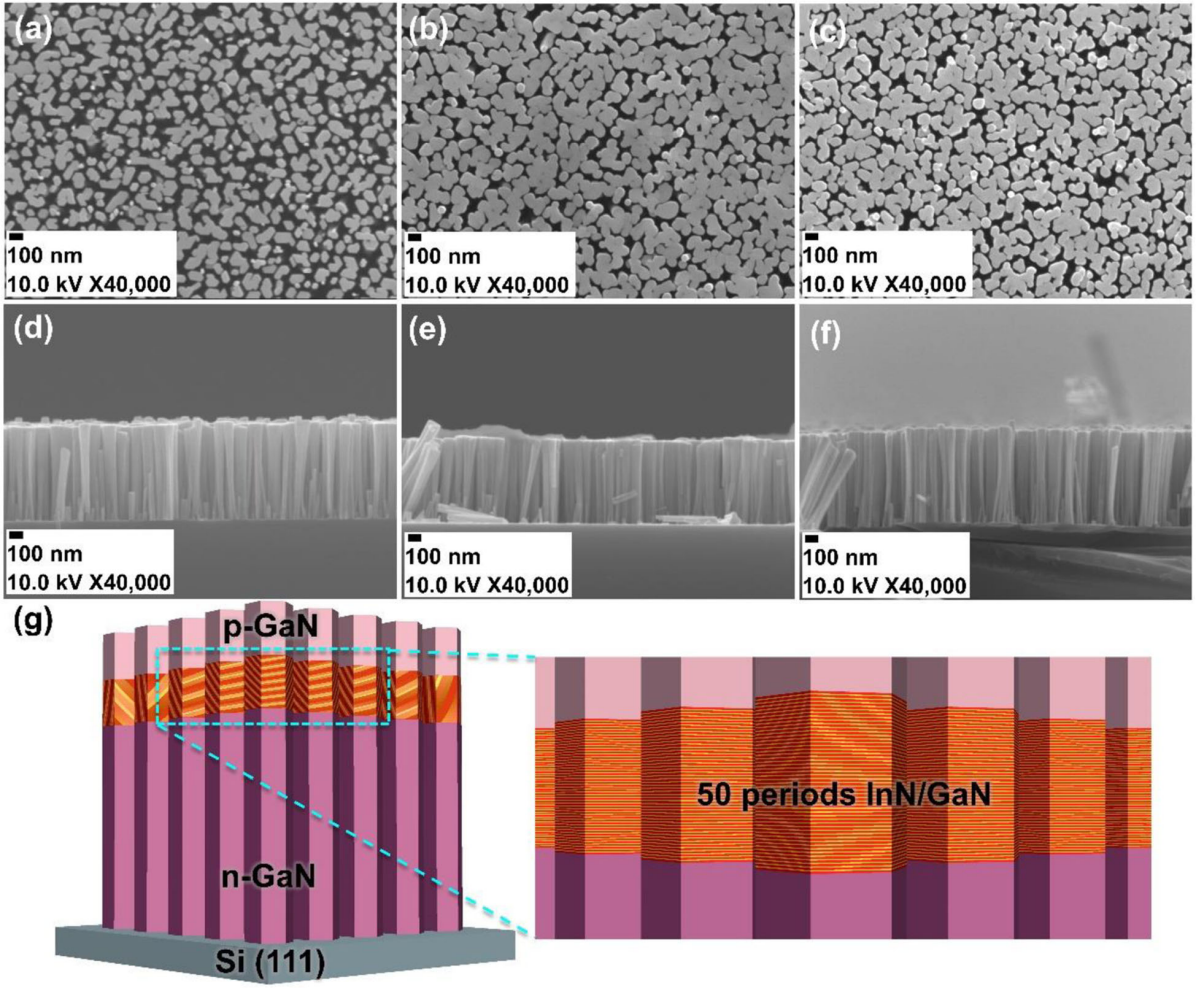
The SEM top views of **a** n-GaN (sample A), p-GaN/InGaN/n-GaN with the In and Ga atoms impinged cycle times of **b** 20 s/30 s (sample B) and **c** 30 s/30 s (sample C). The SEM cross-section views of **d** sample A, **e** sample B, and **f** sample C. **g** A schematic diagram of p-GaN/InGaN/n-GaN nanorod structure

The top views and cross-section views of scanning electron microscopy (SEM) images are shown in Fig. 1a–f illustrating the morphology of the as-grown nanorods. From left to right, Fig. 1 a–c represents the variation in the surface morphology of Si:GaN (sample A) and Mg:GaN/InGaN/Si:GaN with varied In/Ga atoms impinging cycle time of 20 s/30 s (sample B) and 30 s/30 s (sample C) during the 50-cycle InGaN growth, respectively. The diameters of Si:GaN and Mg:GaN/InGaN/Si:GaN nanorods are 30–100 nm and 80–150 nm respectively, while the areal densities are ~ 7 × 10^9^ cm^−2^. The cross-section images of the nanorods are shown in Fig. 1d–f and indicate the length of nanorods to be around 700 nm for samples A to C. The schematic structure of the Mg:GaN/InGaN/Si:GaN samples is shown in Fig. 1g.

**Fig. 2:**
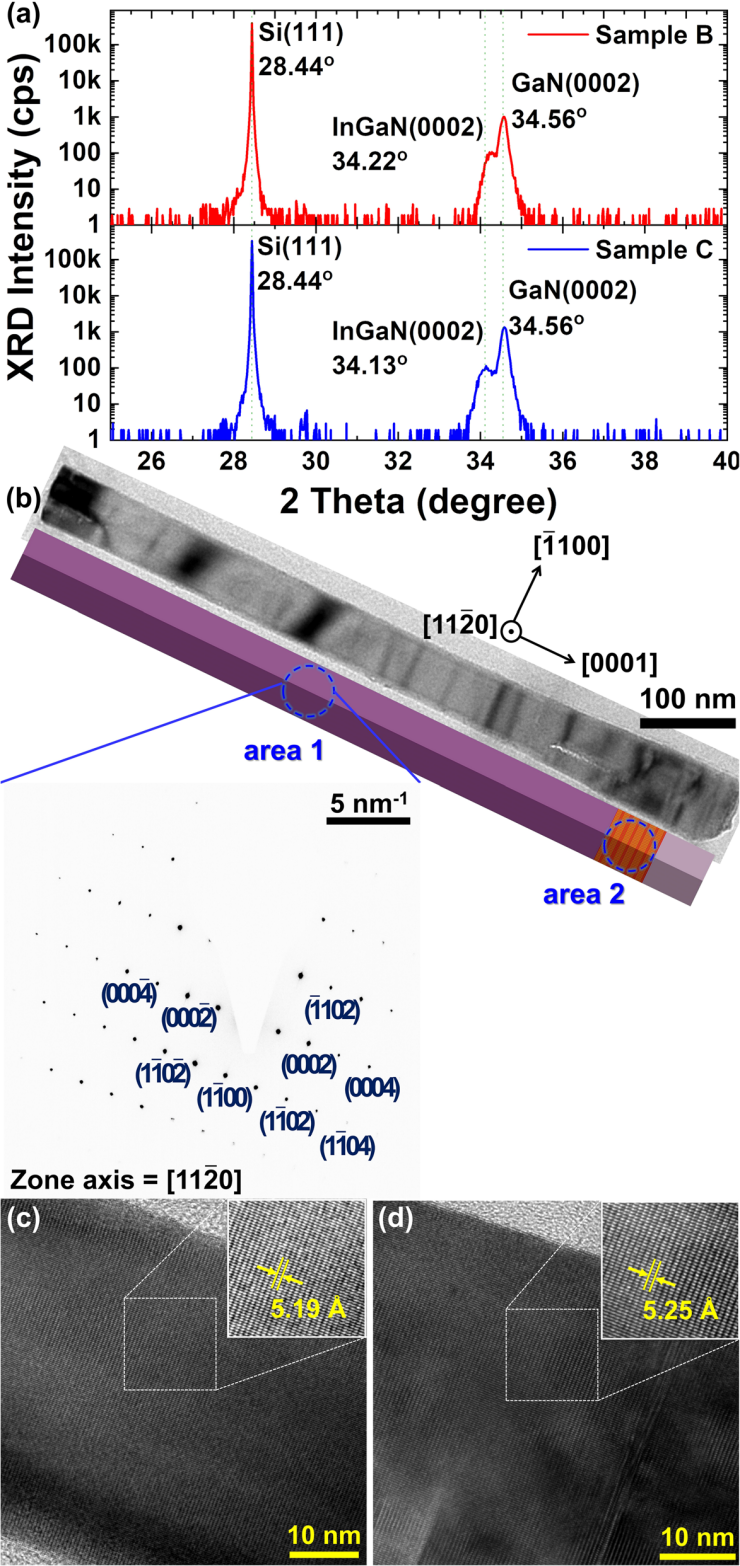
**a** HR-XRD spectra for the theta-2theta scans. The indium content of the InGaN material was estimated to be 8% for sample B (red curve) and 11% for sample C (blue curve) by using Vegard’s law. **b** The dislocation-free nitride nanorod TEM image and SAED pattern of n-GaN region. The single nanorod schematic diagram beneath the image obeys the structural relative scale. The atomic-resolution TEM images of **c** n-GaN in area 1 and **d** InGaN in area 2 show dislocation free and their *c* lattice constants

Figure 2 a recorded the x-ray theta-2theta diffraction measurement focusing on different source impinging cycle time samples. The strongest peak located at 28.44° originates from the Si substrate. A sharp clear peak at 34.56° corresponds to the GaN (0002) diffraction and indicates good inhibition of phase mixing from the InGaN layer. A peak on the lower 2-theta side of the GaN (0002) peak at 34.22° for sample B and at 34.13° for sample C is InGaN (0002). The *c* lattice constants of InN and GaN are 5.760 Å and 5.185 Å respectively [74]. Following Bragg’s law, the *c* lattice constants of InGaN (0002) are calculated as 5.23 Å for sample B and 5.25 Å for sample C. Importing the *c* lattice constant of InGaN (0002) to Vegard’s law, the In concentration can be estimated as 8% for sample B and 11% for sample C without consideration of strain. Figure 2 b shows the low magnification TEM image of sample C and schematic diagram of its structure. The area 1 and area 2 are n-GaN and InGaN regions respectively. The selective-area electron diffraction (SAED) pattern taken at the area 1 demonstrates that the [0001] direction is parallel to the long axis of the nanorod and a common growth direction of nitride nanorods. Moreover, no dislocation is found in the crystal. In Figure 2 c and d, the atomic-resolution TEM images yield the *c* lattice constants of GaN and InGaN as 5.19 Å and 5.25 Å respectively, the same as the results calculated by Bragg’s law via XRD theta-2theta scan. Moreover, the *c* lattice constant of InGaN for sample B is 5.23 Å via atomic-resolution TEM images shown in the Supplementary Information: Figure S1. In addition, high-angle annular dark-field (HAADF) images and energy dispersive x-ray spectroscopy (EDS) line scan, indicating the indium distribution, are included in Supplementary Information: Figure S2.

### Electrical and Optical Characteristics Analysis

**Fig. 3:**
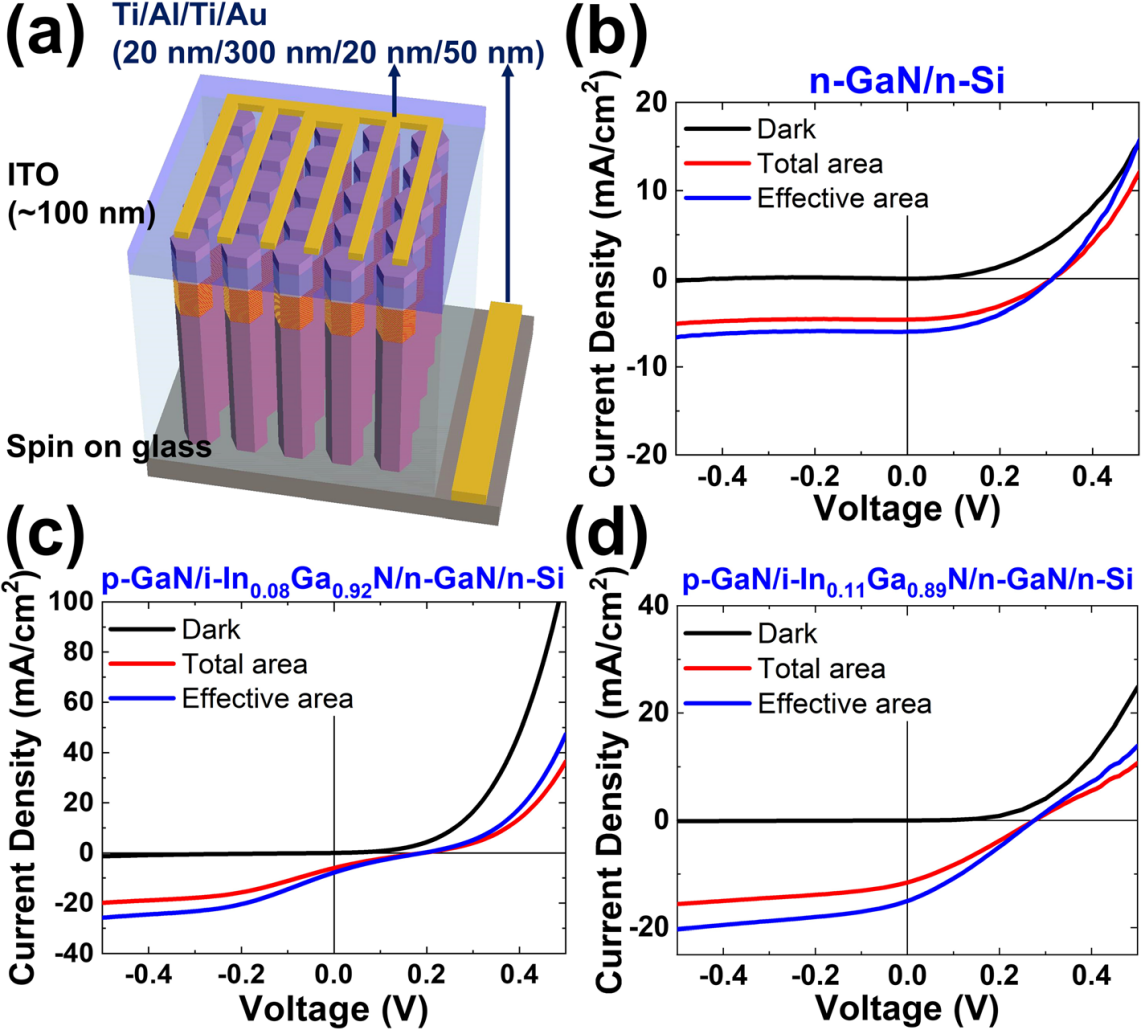
**a** The nanorod assemble solar cell schematic diagram. The current density-voltage curves of **b** n-GaN/n-Si, **c** p-GaN/In_0.08_Ga_0.92_N/n-GaN/n-Si, and **d** p-GaN/In_0.11_Ga_0.89_N/n-GaN/n-Si nanorod assemble solar cells measured under 1 sun, AM 1.5G solar simulator

The current density versus voltage measurements were performed by a Keithley 2400 source meter. Figure 3 a displays the nanorod assemble solar cell schematic diagram. The total device area is 0.12 mm^2^ and the effective area under the illumination excluding the contact metal is 0.0924 mm^2^. To collect photon-generated electrons, a 100-nm transparent conductive ITO film is deposited on the top of the p-GaN to connect the nanorods and the Ti/Al/Ti/Au (20 nm/300 nm/20 nm/50 nm) finger electrode. Photoelectric characteristic analyses of the device were also conducted with solar simulator under 1 sun, AM 1.5G condition as shown in Fig. 3b–d. The series resistance *R*_s_ values determined from Fig. 3b–d are 83 Ω, 250 Ω, and 2.5 kΩ and the shunt resistance *R*_sh_ values are 413 kΩ, 550 kΩ, and 2 MΩ for samples A, B, and C respectively. The photocurrent density at zero voltage, J_sc_, of In_0.08_Ga_0.92_N device (sample B) and In_0.11_Ga_0.89_N device (sample C) is 7.77 mA/cm^2^ and 14.96 mA/cm^2^ respectively. The photocurrent enhancement over the increasing In concentration was demonstrated via J_sc_ comparison. Furthermore, Krogstrup et al. illustrated that the light-concentrating property in nanorod solar cells can enhance light absorption and provide high photocurrent [64]. The open-circuit voltage (V_oc_) and fill factor (FF) of sample C are 0.28 V and 30% respectively. Several groups also demonstrated nanorod structures with lower V_oc_ [72, 75, 76]. To elucidate the real photovoltaic performance in an actually illuminated area, effective-area PCE, PCE_eff_, establishes an efficiency based on the effective area which excludes the grid electrode area, while total-area PCE, PCE_tot_, considers the whole device area. It is notable that the PCE_tot_ and PCE_eff_ values are 0.98% and 1.27% which indicate a higher PCE of nitride nanorod solar cell ever reported. The main contribution comes from the high J_sc_, although the V_oc_ is lower than other III-nitride nanorod solar cell [65, 77]. There are two possible reasons of low V_oc_, including the quasi-Fermi level limited at the p-n Si junction that Si bandgap is 1.12 eV based on the band diagram and a confined current path may be created due to surface Fermi level pinning [66]. Table 1 summarizes J_sc_, V_oc_, FF, and PCE comparison of three samples.

**Table 1:** J_sc_, V_oc_, FF, and effective-area PCE comparison of three samples

J_**sc**_ (mA/cm^**2**^)	V_**oc**_ (V)	FF (%)	PCE (%)
**n-GaN/n-Si (sample A)**			
5.98	0.25	35	0.52
**p-GaN/i-In**_**0.08**_**Ga**_**0.92**_**N/n-GaN/n-Si (sample B)**			
7.77	0.19	21	0.31
**p-GaN/i-In**_**0.11**_**Ga**_**0.89**_**N/n-GaN/n-Si (sample C)**			
14.96	0.28	30	1.27

**Fig. 4:**
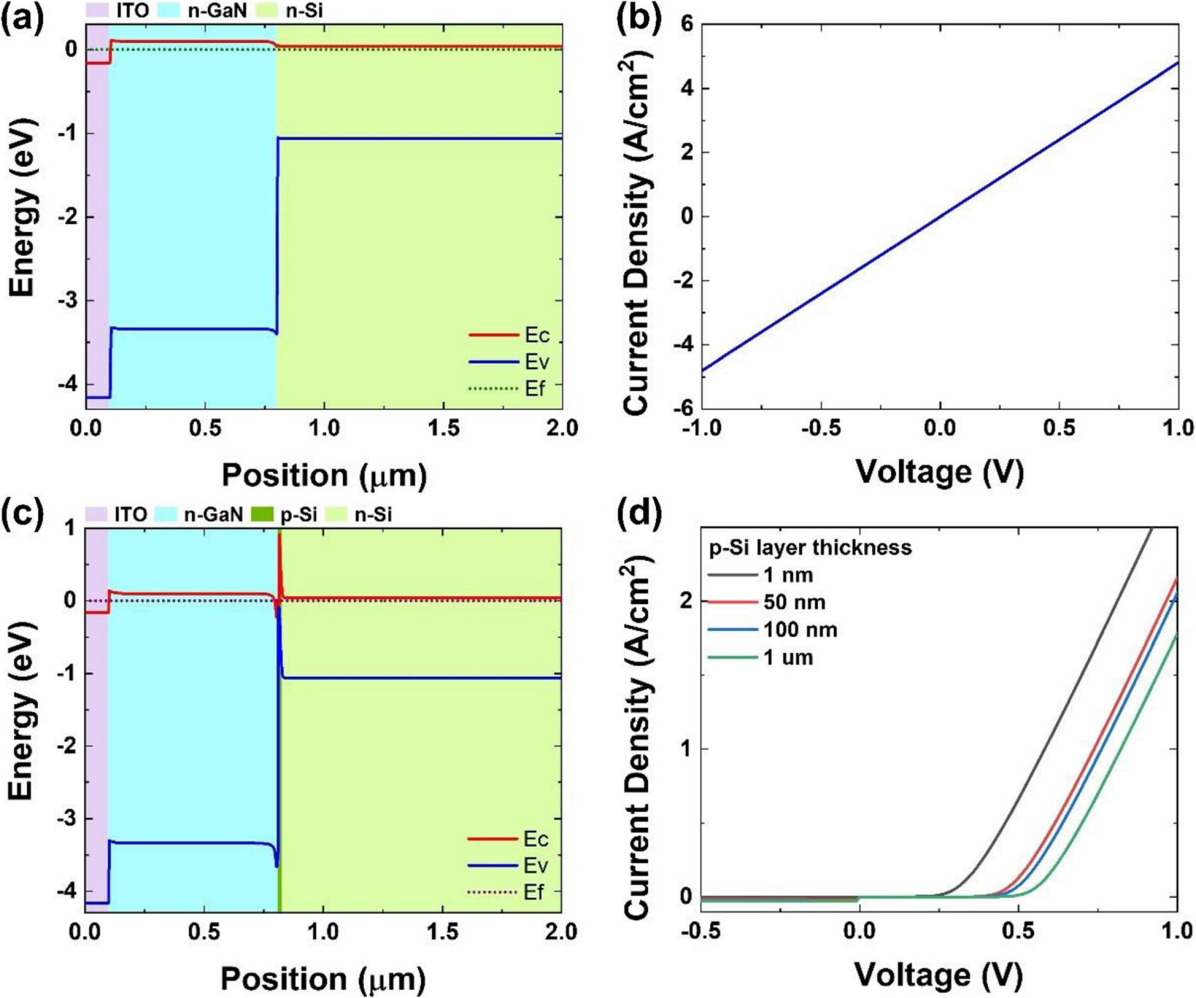
**a** ITO/n-GaN/n-Si band diagram, **b** ITO/n-GaN/n-Si J-V curve, **c** ITO/n-GaN/p-Si/n-Si band diagram, and **d** J-V curves of ITO/n-GaN/p-Si/n-Si simulated by 1D-DDCC program

To understand the physical and electrical properties, the band diagrams are calculated by using a 1D-DDCC (One Dimensional Poisson, Drift-Diffusion, and Schrodinger Solver) program [78]. The electron affinities of ITO, Si, and GaN used are 4.40 eV, 4.05 eV, and 4.1 eV respectively. Figure 4 a and b shows the ITO/n-GaN/n-Si band diagram without voltage bias and J-V curve under dark respectively. It illustrates that the ITO/n-GaN/n-Si structure does not have rectifying effect and shows a linear J-V profile. The potential barrier of the hetero-interface can be ignored for carriers to transport because the conduction band offset between Si and GaN is expected to be a small value of 50 meV. This resistor-like linear J-V curve is in contradiction to the experimental results.

**Fig. 5:**
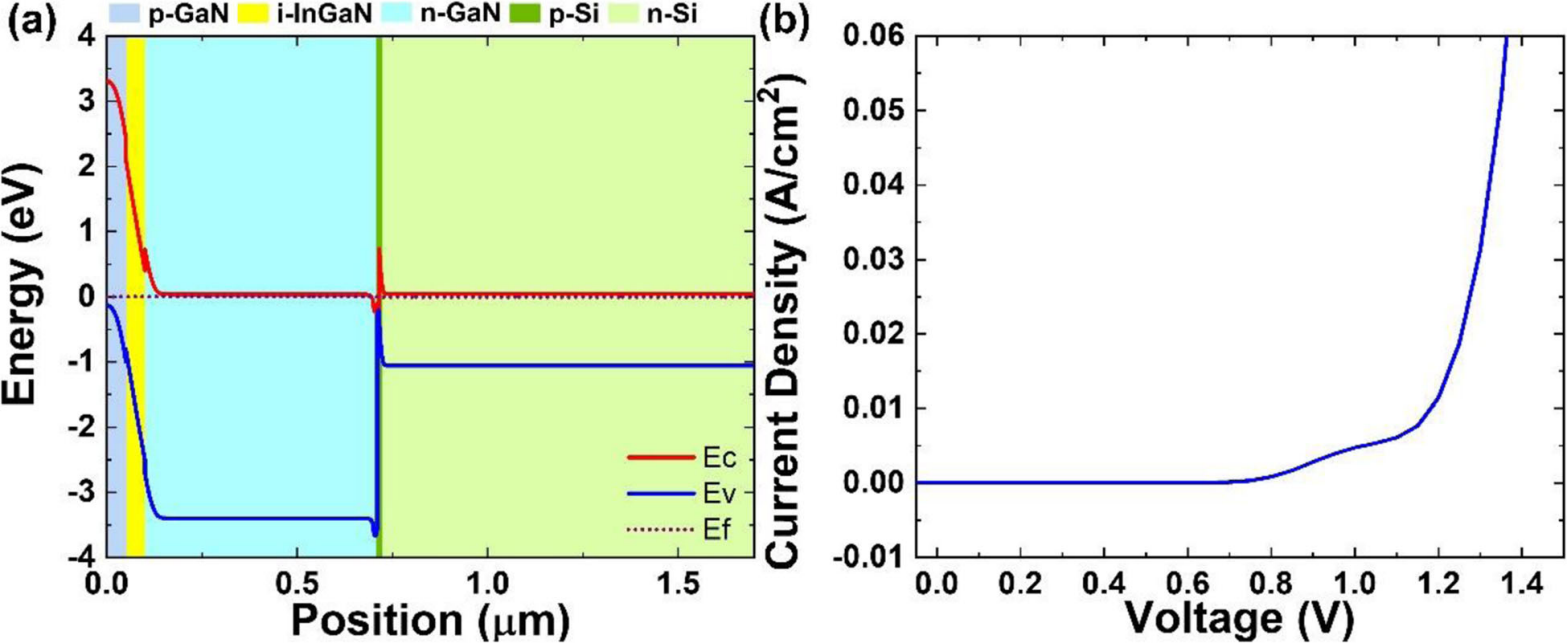
**a** The p-i-n nitride nanorod on p-n Si solar cell band diagram simulated by 1D-DDCC program. **b** J-V curve simulation of p-i-n nitride nanorod on p-n Si solar cell

A possible explanation of J-V curve results is that Ga diffusion induces a p-Si at the GaN/Si interface and creates a p-n junction. Reichertz et al. [14] and Neplokh et al. [76] have verified the Al diffusion into the silicon substrate during the growth of nitride layers. Boron, Al, and Ga are IIIB group elements which can be a dopant for p-Si layer formation. However, Ga diffusion rate is 8 nm/day at 700 °C [79]. Figure 4 c shows a band diagram which includes a very thin (1 nm) p-Si layer between n-GaN and n-Si interface. A small built-in electric field is created in p-n Si junction that can drive the electrons to n-Si substrate and holes to ITO contact layer. The thickness-dependent J-V curves demonstrate that the diode turn-on voltage decreases when the thickness of p-Si layers becomes thinner in Fig. 4d. The ultra-thin p-Si will be a limitation for quasi-Fermi level separation and reduce the V_oc_ of solar performance. The simulated electrical property with the p-Si layer included is closer to this research result. Therefore, the band diagram of Mg:GaN/InGaN/Si:GaN/p-Si/n-Si structure can be built as a model as in Fig. 5. Illumination of AM 1.5G light from the top leads to the absorption of photons with energy higher than InGaN bandgap. When light irradiates onto the Si through the rods and the interspace between the rods, the photons with energy larger than Si bandgap could also be absorbed by the p-n Si substrate and photocurrent is produced. Simultaneously, the electron-hole pairs generated in the nitride regions by the short-wavelength light are separated by p-i-n junction built-in electric field. In the end, photo-generated carriers could be collected through the top indium tin oxide (ITO) contact to Mg:GaN and the bottom Si n-contact. Based on this structure model and considering Schottky barrier introduced by ITO contact, the J-V curve simulation is shown in Fig. 5b. The simulated J-V curve indicates that S-shape is caused by non-ohmic behavior of the p-contact. That is a possible reason to explain the S-shape existed for Mg:GaN/u-InGaN/Si:GaN (samples B and C) in Fig. 3 c and d. Therefore, the current densities at negative bias (J_negative bias (− 0.5 V)_) where the S-shape is flattened are noted in Table S2. J_negative bias_ can be a checking point for further optimization and a targeted value.

**Fig. 6:**
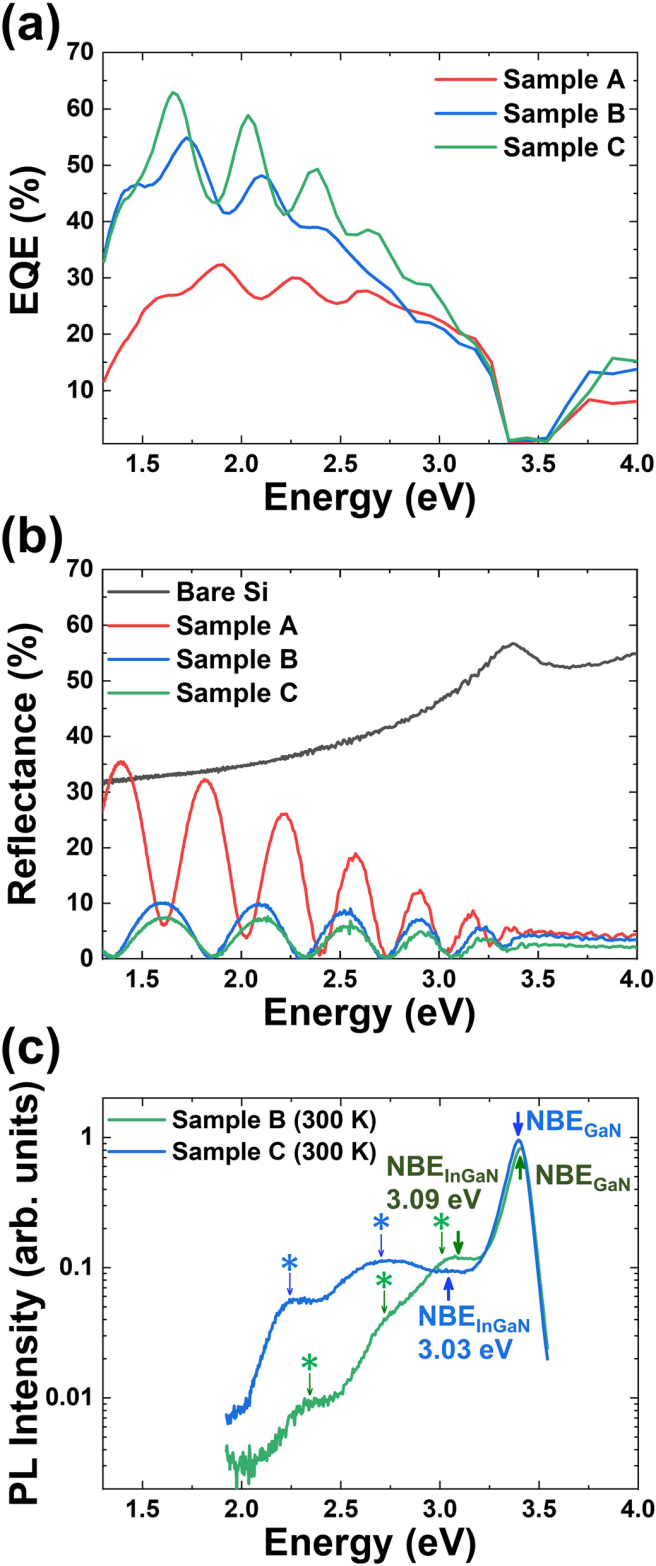
**a** External quantum efficiency spectra of three nitride nanorods/Si samples. **b** The reflectance spectra of bare Si wafer and three nitride nanorods/Si samples. **c** The room temperature photoluminescence spectra of two InGaN samples

The EQE measurement results without light bias (additional unmodulated light) are shown in Fig. 6a, which compares the EQEs of Si:GaN/n:Si (sample A), Mg:GaN/u-In_0.08_Ga_0.92_N/Si:GaN (sample B), and Mg:GaN/u-In_0.11_Ga_0.89_N/Si:GaN (sample C). Sample C has higher In concentration which might be responsible for the higher values at short wavelength due to the absorption in the InGaN layer. The maximum EQEs in samples A, B, and C are 32%, 55%, and 63% respectively. Compared with the reflectance spectra of Si wafer, samples A, B, and C shown in Fig. 6b, the oscillations of EQEs and reflectance spectra are due to the interference from different layers. The bare Si wafer has the highest reflectance due to its flat surface. Samples A, B, and C have lower reflectance because nanorod structures have a light trapping effect. Sample C is found to have the highest EQE at long wavelength and the lowest reflectance due to the highest light trapping effect. This result can explain the higher photocurrent generated in sample C. The room temperature (RT, 300 K) photoluminescence (PL) spectra of sample B and sample C are shown in Fig. 6c. The highest peak located at the 3.40 eV is GaN near band edge (NBE) emission. The peaks located at 3.09 eV and 3.03 eV are due to In_0.08_Ga_0.92_N and In_0.11_Ga_0.89_N NBE emission. The results are similar to the values from the bowing equation calculation of 3.1 eV and 3.0 eV at RT [4]. It also shows the same strong Fabry-Perot oscillations (marked by star signs) as the EQEs and the reflectance spectra, representing the smooth interfaces between each layer/surface.

## Conclusion

High quality Mg:GaN/InGaN/Si:GaN and Si:GaN nanorods grown on n-Si by plasma-assisted molecular beam epitaxy are successfully demonstrated. Photovoltaic measurements exhibit a PCE_eff_ of 1.27% and a PCE_tot_ of 0.98% under 1 sun, AM 1.5G illumination for Mg:GaN/u-In_0.11_Ga_0.89_N/Si:GaN which has a higher In concentration and a higher light trapping effect inducing a high photocurrent. Although Si:GaN nanorods on n-Si device may not have a prominent p-n junction built-in field, the design of a proper heterojunction structure helps to drive the photocarriers to the top and bottom contacts and enhances the cell performance.

## Supplementary information

**Additional file 1: Table S1.** Comparison of the performance of III-nitride nanorods solar cells. **Table S2.** The comparison of photocurrent density at negative biases where the S-shape is flattened. **Figure S1.** The atomic-resolution TEM images of InGaN for In_0.08_Ga_0.92_N (Sample B) show dislocation free and lattice constant *c*. **Figure S2.** (a) The HAADF image of Sample C. (b) EDS line scan of a single nanorod.

## Data Availability

The authors declare that the materials and data are available to the readers, and all conclusions made in this manuscript are based on the data which are all presented and shown in this paper.

## Abbreviations

LED: Light-emitting diode
PCE: Power conversion efficiency
J_sc_: Short-circuit current density
PA-MBE: Plasma-assisted molecular beam epitaxy
HR-XRD: High-resolution x-ray diffraction
HR-TEM: High-resolution transmission electron microscopy
EQE: External quantum efficiency
BEP: Beam equivalent pressure
MME: Metal modulated epitaxy
ITO: Indium tin oxide
V_oc_: Open-circuit voltage
RT: Room temperature
PL: Photoluminescence
NBE: Near band edge
