# Supplementary material for: Light Trapping Induced High Short-Circuit Current Density in III-Nitride Nanorods/Si (111) Heterojunction Solar Cells
Source: Nanoscale Res Lett. 2020 Aug 20;15:167. doi: 10.1186/s11671-020-03392-z (PMC7441121; doi:10.1186/s11671-020-03392-z)
Supplement: Supplementary file 1 — Additional file 1: Table S1. Comparison of the performance of III-nitride nanorods solar cells. Table S2. The comparison of photocurrent density at negative biases where the S-shape is flattened. Figure S1. The atomic-resolution TEM images of InGaN for In0.08Ga0.92N (Sample B) show dislocation free and lattice constant c. Figure S2. (a) The HAADF image of Sample C. (b) EDS line scan of a single nanorod. [file 11671_2020_3392_MOESM1_ESM.pdf]

Supplementary Information

Light trapping induced high short-circuit current density in III-nitride nanorods/Si (111) heterojunction solar cells

Ching-Wen Chang[1, 2, 3], Paritosh V. Wadekar[1], Hui-Chun Huang[4], Quark Yung-Sung Chen[1, 5], Yuh-Renn Wu[6], Ray T. Chen[2], and Li-Wei Tu[1, 7*]

[1]Department of Physics and Center of Crystal Research, National Sun Yat-Sen University, Kaohsiung 80424, Taiwan, ROC.

[2]Department of Electrical and Computer Engineering, The University of Texas at Austin, Austin, TX 78758, USA.

[3]Research Center for Applied Sciences, Academia Sinica, Taipei 11529, Taiwan, ROC.

[4]Department of Materials and Opto-electronic Science, National Sun Yat-Sen University, Kaohsiung 80424, Taiwan, ROC.

[5]Department of Physics and Texas Center for Superconductivity, University of Houston, Houston, TX 77004, USA.

[6]Institute of Photonics and Optoelectronics and Department of Electrical Engineering, National Taiwan University, Taipei 10617, Taiwan, ROC.

[7]Department of Medical Laboratory Science and Biotechnology, Kaohsiung Medical University, Kaohsiung 80708, Taiwan, ROC.

*Corresponding author: lwtu@mail.nsysu.edu.tw

1. The recent nanorod/nanowire photovoltaic researches comparison

Table S1 Comparison of the performance of III-nitride nanorods solar cells

Reference no.	Device type	J_{sc} (mA/cm^2)	V_{oc} (V)	FF (%)	PCE (%)
[31]	PIN InN/n-Si	14.36	0.14	34	0.68
[65]	core–shell InGaN/GaN MQWs	1	0.5	54	0.3
[66]	PIN InGaN/n-Si	4.6	0.22	34	0.5
[75]	core–shell InGaN/GaN MQWs single nanorod	0.60	0.95	83	0.33
[76]	n-GaN/p-n Si	-	0.36	20	2.7
[77]	Coaxial and uniaxial InGaN/GaN MQWs	2.28	0.73	70	1.16
This work	PIN InGaN/n-Si	14.96	0.28	30	1.27/0.98

2. TEM images of $In_{0.08}Ga_{0.92}N$ (Sample B)

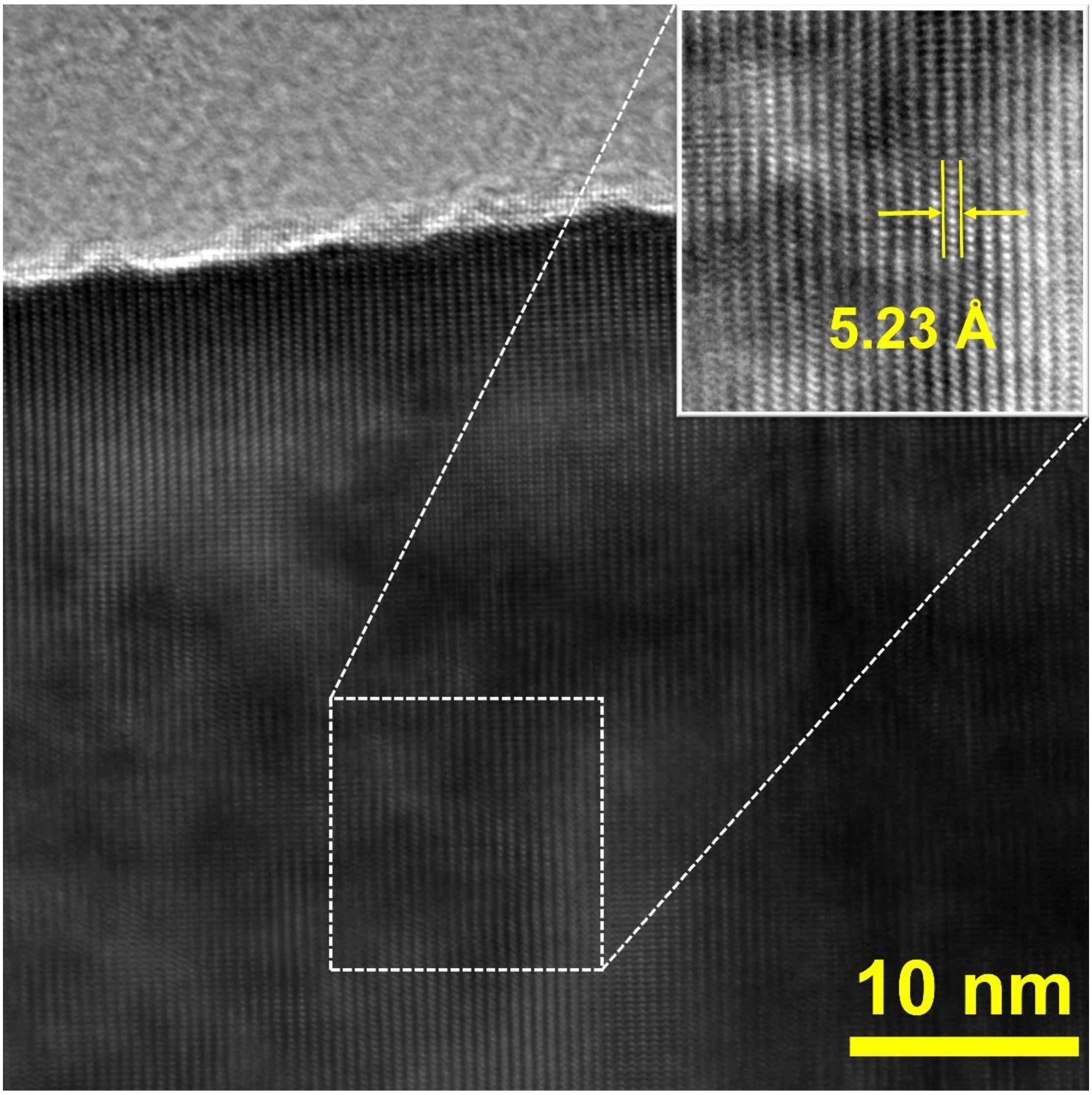

Figure S1 The atomic-resolution TEM images of InGaN for $In_{0.08}Ga_{0.92}N$ (Sample B) show dislocation free and lattice constant *c*.

3. HAADF images and EDS of Sample C

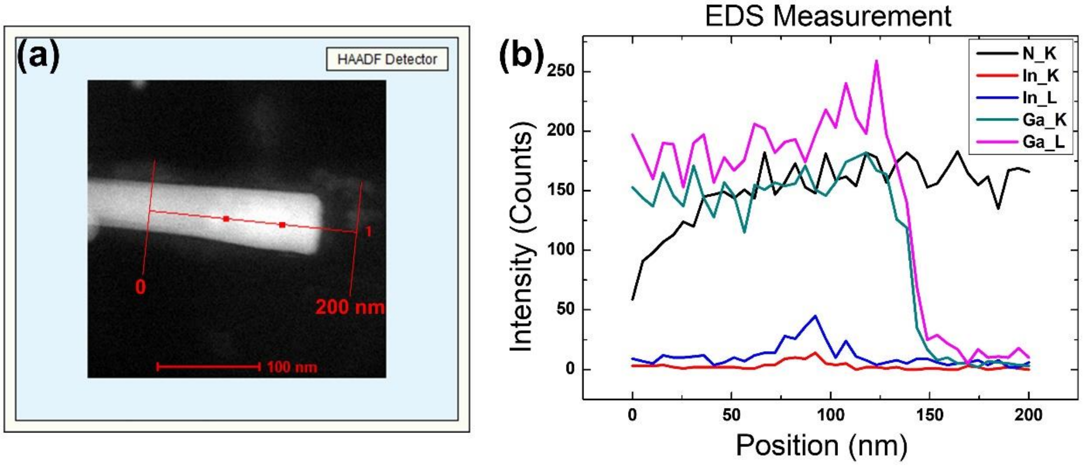

Figure S2 (a) The HAADF image of Sample C. (b) EDS line scan of a single nanorod.

4. The comparison of photocurrent density at negative biases where the S-shape is flattened

Sample	$J_{negative\ bias\ (-0.5\ V)}$ (mA/cm^2)
n-GaN/n-Si (Sample A)	6.6
p-GaN/i-$In_{0.08}Ga_{0.92}N$/n-GaN/n-Si (Sample B)	25.8
p-GaN/i-$In_{0.11}Ga_{0.89}N$/n-GaN/n-Si (Sample C)	20.3
